# Evaluation of the COVID-19 vaccine effectiveness on the outcomes of COVID 19 disease in Iran: a test-negative case-control study

**DOI:** 10.3389/fimmu.2024.1420651

**Published:** 2024-08-20

**Authors:** Fatemeh Khosravi Shadmani, Ghobad Moradi, Mohammadreza Naghipour, Fatemeh Torkaman Asadi, Ali Ahmadi, Alireza Mirahmadizadeh, Ali Akbar Haghdoost, Bita Mesgarpour, Seyed Mohsen Zahraei, Mohammad Mehdi Goya, Majid Mokhtari, Roya Safari-Faramani, Fariba Zomorrodi Zare, Maryam Chegeni, Farid Najafi

**Affiliations:** ^1^ Research Center for Environmental Determinants of Health (RCEDH), Health Institute, Kermanshah University of Medical Sciences, Kermanshah, Iran; ^2^ Social Determinants of Health Research Center, Research Institute for Health Development, Kurdistan University of Medical Sciences, Sanandaj, Iran; ^3^ Gastrointestinal and Liver Diseases Research Center, Guilan University of Medical Sciences, Rasht, Iran; ^4^ Infectious Disease Research Center, Hamadan University of Medical Sciences, Hamadan, Iran; ^5^ Department of Epidemiology and Biostatistics, School of Health and, Modeling in Health Research Center, Shahrekord University of Medical Sciences, Shahrekord, Iran; ^6^ Non-Communicable Diseases Research Center, Shiraz University of Medical Sciences, Shiraz, Iran; ^7^ Modeling in Health Research Center, Institute for Futures Studies in Health, Kerman University of Medical Sciences, Kerman, Iran; ^8^ National Institute for Medical Research Development (NIMAD), Tehran, Iran; ^9^ Center for Communicable Disease Control, Ministry of Health and Medical Education, Tehran, Iran; ^10^ Skull Base Research Centre, Loghman Hakim Hospital, Shahid Beheshti University of Medical Sciences, Tehran, Iran; ^11^ Social Development and Health Promotion Research Center, Research Institute for Health, Kermanshah University of Medical Sciences, Kermanshah, Iran; ^12^ Department of Public Health, Khomein University of Medical Sciences, Khomein, Iran

**Keywords:** vaccine effectiveness, COVID-19, Iran, test-negative case-control, hospital admission, death

## Abstract

**Introduction:**

This study measures the COVID-19 vaccine effectiveness (CVE) against hospital admission and severe COVID-19.

**Methods:**

This study is a test-negative case-control design using data from eight provinces in April, 2021 until March, 2022. The individuals were classified as cases and controls based on the results of the RT-PCR test for SARS-CoV-2 and matched based on the timing of the test being conducted as well as the timing of hospital admission. The measure of association was an odds ratio (OR) by univariate and multiple logistic regression. The multiple logistic regression has been carried out to take confounding factors and potential effect modifiers into account. The CVE was computed as CVE = (1 – OR)*100 with 95% confidence interval.

**Results:**

Among 19314 admitted patients, of whom 13216 (68.4%) were cases and 6098 (31.6%) were controls, 1313 (6.8%) died. From total, 5959 (30.8%) patients had received the vaccine in which one, two, and booster doses were 2443 (12.6%), 2796 (14.5٪), and 720 (3.7٪), respectively. The estimated adjusted effectiveness of only one dose, two doses and booter vaccination were 22% (95% CI: 14%-29%), 35% (95% CI: 29%-41%) and 33% (95% CI: 16%-47%), respectively. In addition, the adjusted vaccine effectiveness against severe outcome was 33% (95% CI: 19%- 44%), 34% (95% CI: 20%- 45%) and 20% (95% CI: -29%- 50%) for those who received one, two and booster vaccinations, respectively.

**Conclusion:**

Our study concluded that full vaccination, though less effective compared to similar studies elsewhere, decreased hospital admissions and deaths from COVID-19 in Iran, particularly during the Delta variant period, with an observed decline during the Omicron variant dominance.

## Introduction

Since the announcement of the pandemic of COVID-19 on March 11,2020, and in addition to the recently available effective treatment and all other mitigation strategies, vaccination is still one of the most important interventions, which is a safe, simple, and effective way to protect communities against COVID-19 (1, 2). Since the first emergency use validation certificate for the Pfizer/BioNTech vaccine from WHO’s Advisory Group of Experts on Immunization (WHO timeline), the countries that quickly implemented effective vaccination programs, such as Britain or Israel, reported a significant reduction in the number of confirmed cases, ICU admissions, and deaths (3). The COVID-19 vaccine efficacy analysis addresses the efficiency of various vaccines in controlling the disease under ideal and controlled laboratory conditions in clinical trials. Pre-approval studies, which used randomized clinical trials to measure the efficacy of the vaccines, could be different (low follow-up duration and excluding some populations) from the vaccine effectiveness. Clinical trials have demonstrated the efficacy against symptomatic infection ranging from 95.0% and 94.1% for the Pfizer-BioNTech and Moderna, to 50.7% for the Sinovac (4–6). Vaccine effectiveness in real-world settings differs from trial results. Studies have shown vaccine effectiveness in preventing of infection varies from 47% to 95% for different brands after two doses in under 20 weeks after vaccination (7–9). On the other hand, recent analysis in Qatar found no evidence of protection against infection after 20 weeks post-vaccination (10).

There have been serious concerns about a decrease in the effectiveness of vaccines because of new emerging variants of interest and variants under monitoring, as well as the weaning of antibodies, which in turn show the importance of such studies in different settings (11). Furthermore, no vaccine is 100% effective against COVID-19. There is no evidence that the COVID-19 vaccine can prevent transmission, but it can help protect against the COVID-19 infection. Various countries have reported that the number of new COVID-19 cases and transmission rates have decreased in many areas, possibly due to the protective effects of vaccines and/or restrictions (12–15).

However, vaccine candidates have been evaluated separately, which makes comparing the efficacy of different vaccines challenging. Therefore, it is of high importance to reinvestigate the immunogenicity and safety observed in vaccine trials in other real-world studies, specifically in those vaccines with a low profile of being studied by independent researchers (16).

In addition, the new coming variants of interest raise the question about the effectiveness of vaccines that have been developed based on the old variants. In fact, the purpose of any vaccine is to reduce the COVID-19 incidence and hospitalization and its case fatality. Questions about the performance of vaccines in the real world can only be answered in post-introduction vaccine effectiveness studies.

In Iran, approximately 65 million people have received the first dose, 58 million people have received the second dose, and more than 31 million people have received the booster dose of vaccine as of March 25, 2023 (17). In Iran, different vaccines have been approved by Iranian Food and Drug Organization and other authorities, including: Sputnik light and V, AZD1222, BBIBP-CorV, Bharat Covaxin, COVIranBarekat, FAKHRAVAC, Nora vaccine, Soberana 02, Razi Cov Pars and SpikoGen. The number of different products of vaccines used in Iran and the use of domestic products that have not been approved by the WHO are all characteristics of vaccination programs in Iran, which in turn make this study of high importance to health policy makers.

A few studies on the effectiveness of the COVID-19 vaccine have been conducted in Iran. These studies have shown that the effectiveness of the two-dose COVID-19 vaccine in preventing infection ranges from 72% to 85% (7, 18) and in preventing death ranges from 20% to 55% (7, 19). However, there is a necessity to reexamine the efficacy of vaccines using varied methodologies across distinct temporal phases. The present study is one part of the large EMRO supported study in several countries, which was carried out according to WHO guidelines. The aim of the study is evaluation of the effectiveness of COVID-19 vaccines on the outcomes of COVID-19 disease in Iran.

## Materials and methods

### Study design and setting

This study is a test-negative design (TND) case-control study that is performed following the WHO guidelines. TND is an efficient form of case-control study commonly applied to vaccine effectiveness (VE) estimation (20). In this study, we evaluated the RT-PCR results of SARS-CoV-2 among individuals who have been diagnosed with a severe acute respiratory infection (SARI) and hospitalized in main referral hospitals in provincial/district level.

The study was conducted in collaboration with several main referral hospitals in provincial/district level across Iran, covering a wide geographical range from the north and northeast regions of Shahroud, Guilan and Mazandran to the west (Kermanshah, Kurdistan, and Hamedan), the southern (Fars), and the southwestern (Charmahal and Bakhtiari). The selected hospitals affiliated with each university were well-equipped with specialists in infectious diseases, ICU care, and other relevant fields and facilities to effectively handle COVID-19 patients. For this study, we included individuals aged over 12 years who were hospitalized with SARI symptoms in a hospital participating in the SARI surveillance in specific provinces.

### Data sources

For the purpose of this study, we mainly used two datasets: MCMC and Integrated Health Record System (locally known as SIB). The MCMC dataset contained all relevant data regarding hospital admissions, including signs, symptoms, and other clinical findings. The SIB dataset is based on the electronic health record system in Iran, which is designed to consolidate health information of individuals and document primary healthcare services along with other health services offered by rural and urban health centers. This database includes essential information on COVID-19 vaccination, including the vaccine brand and the dates of administration for each dose. All participating provinces were asked to link these two datasets using national IDs. The central team held two workshops to ensure consistency in the linking process.

### Study procedures, period and recruitment

**Figure 1:**
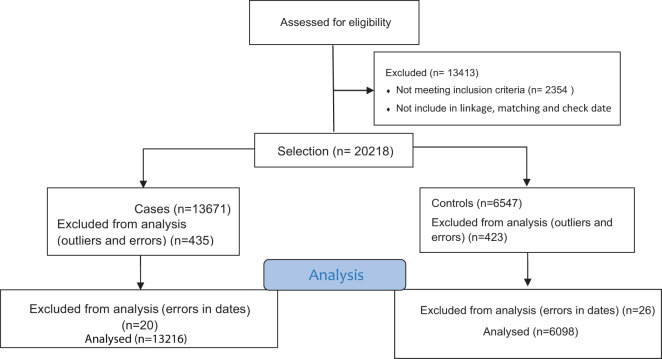
Selection method of case and control.

Study protocol confirming by EMRO and research ethics committees of Kermanshah University of Medical Sciences. After the linkage of registering data, cases and controls were determine and matched based on the hospital and time of hospital admission, ensuring that the hospital admission dates of cases and controls were no more than two weeks apart. The entire data collection process lasted for 5 months. The selection methods of case and control are shown in **Figure 1**.

For the purpose of this study, we decided to include all SARI hospitalized cases (test-positive and test-negative patients) from April 30, 2021 (when the number of vaccination doses administered reached 2 million) until March 20, 2022 (when Delta and Omicron variants were predominant), if they meet all other inclusion criteria. The dominant variant of circulation was categorized as Delta (from 30th April 2021 to 31st December 2021) and Omicron (from 1st January 2022 until 20th June 2022).

### Variables definition

A SARI patient has been defined using the WHO SARI case definition as a hospitalized person with acute respiratory infection with a history of fever or measured fever of ≥ 38°C and cough with symptom onset within the last 10 days (21). A confirmed COVID-19 case defined as a patient hospitalized with syndromic surveillance for SARI symptoms with a respiratory sample positive for SARS-CoV-2 by RT-PCR, either within 48 hours of hospital admission or documented within 14 days before hospital admission. The control group is defined as patients hospitalized with SARI symptoms with a respiratory sample negative for SARS-CoV-2 by RT-PCR on hospital admission, up to 48 hours after or the period of 14 days prior to hospital admission (20).

For each SARI patient, details such as the dates of vaccine administration, type of vaccine, brand name, and batch code for every dose of the COVID-19 vaccine were recorded. This information was gathered through the SIB and MCMC systems. Vaccines from any brand were included in the study and classified into three groups: BBIBP-CorV, AZD1222, and others. If a SARI patient did not receive the COVID-19 vaccine or if the vaccination occurred within 14 days before symptom onset, the patient was considered unvaccinated. Partially vaccinated refers to an individual who received one dose of a specific COVID-19 vaccine if they have received that dose more than 14 days before the onset of symptoms related to SARI. Fully vaccinated patients were considered fully vaccinated if they had received the second (final) dose, at least 14 days before symptom onset. Patients considered fully vaccinated with a booster dose if they received the second dose with one booster dose at least 7 days before symptom onset.

### Inclusion and exclusion criteria

All SARI cases defined by current country protocols for SARI surveillance were part of the target group if they were eligible for the COVID-19 vaccine on the date of hospital admission. Those who were not eligible for COVID-19 vaccine at the time of hospital admission (i.e., those who are less than 12 years old); could not be swabbed due to severe septum deviation, obstruction or other conditions that contra-indicate swabbing; had a history of hospitalization within the 14 days prior to this admission (including transfers from another hospital), were excluded from the study. In this study, we exclude patient self-report as the only source of information on vaccination status and vaccine details. In addition, individuals who received their second dose within less than a 3-month interval and the booster dose within less than a 1-month interval were not included in the study.

### Statistical analyses

Using the test-negative design, the measure of association was an odds ratio (OR) calculated by univariate and multiple logistic regression. The multiple logistic regression has been carried out to take confounding factors and potential effect modifiers into account. The model for vaccine effectiveness against hospital admission was adjusted for age, sex, and contact history with a COVID-19 patient, onset date of first symptoms, and time period longer than six months after vaccination. This provided the adjusted ORs from which the CVE was computed as CVE = (1 – OR)*100. A 95% confidence interval computed around the point estimate.

Because the chronic condition had a strong relationship with age and recorded chronic condition was self-reported, we only added age to the models. In addition, in order to overcome the low sample size for death and ICU admission, we define the severe outcome as those experiencing ICU admission and/or death. The model for vaccine effectiveness against severe outcome was adjusted for age, sex, province, length of hospital stays, history of contact with a COVID-19 patient, type of hospital, time period longer than six months after vaccination and onset week. In addition, stratified analyses (by age group and brand of vaccine) were done to better understand potential effect modifiers and confounders.

## Results

After applying the exclusion criteria, 19,314 individuals were included in the study. Of these, 13,216 (68.4%) were in the case group and 6,098 (31.6%) were in the control group. Among them, there were more women than men (53.5% and 51.2% in the case and control groups, respectively). From total, 28.4% aged ≥ 65 years old. In the case group, 20.0% were admitted to the ICU and 3.9% required mechanical ventilation, while in the control group, the corresponding values were 20.3% and 3.8%, respectively. Among 19,314 admitted patients, 1313 (6.8%) died, of whom 984 (7.4%) were cases and 329 (5.4%) were controls. Overall, 71.4% of the patients had Contact history of patient with COVID-19 cases, and 2.4% of them had previously been infected with COVID-19. From total, 73.1% had positive lung computer tomography. Among all hospitalized patients, 88.2% of them were exposed with the Delta variant, and 11.8% were exposed with the Omicron variant. From total, 41.1% presented with respiratory distress. The other characteristics of the case and control groups are presented in **Table 1**.

**Table 1:** Characteristics of cases (n=13216) and controls (n=6098), April 30, 2021 to June 20, 2022, for Iran is among SARI patients.

Variables		Number of laboratory-confirmed COVID-19 cases/total 13216 (68.4%)	Number of test-negative Controls/total 6098 (31.6%)	P value	Total 19314 (100%)
**Age groups**	**12-44**	4720(35.7)	2081 (34.1)	<0.001	6801 (35.2)
	**45-64**	4950 (37.5)	2081 (34.1)		7031 (36.4)
	**>=65**	3546 (26.8)	1936 (31.8)		5482 (28.4)
**Age, median (IQR)-Yr**	** **	52 (39-65)	54 (40-69)	<0.001	53 (39-66)
**Sex**	**Female**	7072 (53.5)	3120 (51.2)	0.002	10192 (52.8)
	**Male**	6144 (46.5)	2978 (48.8)		9122 (47.2)
**Healthcare worker**	** **	122 (0.9)	47 (0.8)	0.291	169 (0.9)
**Days between onset of symptoms and swabbing**	**1**	267 (2.0)	241 (4.0)	<0.001	508 (2.6)
	**2**	1559 (11.8)	1040 (17.1)		2599 (13.5)
	**3**	1659 (12.6)	978 (16.0)		2637 (13.7)
	**4**	1700 (12.9)	823 (13.5)		2523 (13.1)
	**5**	1621 (12.3)	736 (12.1)		2357 (12.2)
	**6**	1137 (8.6)	411 (6.7)		2548 (8.0)
	**7**	2523 (19.1)	959 (15.7)		2482 (18.0)
	**8**	984 (7.5)	323 (5.3)		2307 (6.8)
	**9**	534 (4.0)	195 (3.2)		2729 (3.8)
	**10**	1232 (9.3)	392 (6.4)		2624 (8.4)
**Admitted to ICU**	** **	2650 (20.1)	1236 (20.3)	0.726	3886 (20.1)
**Mechanical ventilation**	** **	519 (3.9)	232 (3.8)	0.682	751 (3.9)
**Outcome**	**Discharge**	12232 (92.6)	5769 (94.6)	<0.001	18011 (93.2)
** **	**Death**	984 (7.4)	329 (5.4)		1313 (6.8)
Comorbidity*	**0**	9202 (69.6)	4088 (67.0)		13290 (68.8)
	**1**	2501 (18.9)	1172 (19.2)	<0.001	3673 (19.0)
	**>=2**	1513 (11.5)	838 (13.7)		2351 (12.2)
**the patient has previous admitted for COVID19**	** **	85 (0.6)	53 (0.9)	0.083	138 (0.7)
**Contact history of patient with COVID-19 cases**	** **	9681 (73.2)	4115 (67.5)	<0.001	13796 (71.4)
**Previous history of COVID-19**	** **	361 (2.7)	109 (1.8)	<0.001	470 (2.4)
**Positive CT Scan^^1^^**	** **	9806 (74.2)	4310 (70.7)	<0.001	14116 (73.1)
**Pregnant female**	** **	209 (3.0)	88 (2.8)	0.709	297 (2.9)
**Hospital stays, median (IQR)**	** **	5 (4-6)	4 (3-6)	<0.001	5 (3-6)
**O2 saturation**	**<90**	4520 (34.2)	1933 (31.7)	0.001	6453 (33.4)
	**>=90**	7305 (55.3)	3140 (51.5)		10445 (54.1)
	**Missing**	1391(10.5)	1025 (16.8)		2416 (12.5)
**Respiratory Distress**	** **	5436 (41.1)	2427 (39.8)	0.080	7863 (40.7)
**Variant (period of time of variant circulation)**	**Delta (from 2021/30/4 to 2021/31/12)**	11768 (89.0)	5272 (86.4)	<0.001	17040 (88.2)
	**Omicron (from 2022/1/1 to 2022/20/6)**	1448 (11.0)	826 (13.5)		2274 (11.8)

*Cancer, Chronic cardiac disease except hypertension, Hypertension, chronic kidney disease, Chronic liver disease, Chronic respiratory disease, Asthma, Diabetes, HIVAIDS, Neurological disease.

1 Computed Tomography (CT) Scan.

Out of the total, 5,959 (30.8%) patients had been vaccinated, with 63% of them belonging to the case group (**Table 2**). Specifically, 2,443 (12.6%) received one dose, 2,796 (14.5%) received two doses, and 720 (3.7%) received a booster dose. From total of participants with two doses of vaccination, only 0.5% of participants received a heterologous second dose of vaccine. The corresponding value for the booster vaccination was 9.8%. In addition, 61.4% of the individuals who had received two doses of a homologous vaccine were in the case group. Further details on vaccination characteristics are outlined in **Table 2** for both the case and control groups.

**Table 2:** Comparison of vaccination status between cases and controls by vaccine brand and time interval between doses.

Variables	Category	Number of laboratory-confirmed COVID-19 cases/total 13216 (68.4%)	Number of test-negative Controls/total 6098 (31.6%)	Total 19314 (100%)
**Vaccine status**	Un vaccinated	9451 (71.5)	3904 (64.0)	13355 (69.2)
	Partially vaccinated	1587 (12.0)	856 (14.0)	2443 (12.6)
	Fully vaccinated	1717 (13.0)	1079 (17.7)	2796 (14.5)
	Booster	461 (3.5)	259 (4.3)	720 (3.7)
**Vaccine combination in fully vaccinated**	Homologous	1711 (61.4)	1073 (38.6)	2784 (99.5)
	Heterologous	7 (44.4)	6(56.6)	13 (0.5)
**Vaccine combination (booster dose)**	Homologous	419 (90.9)	230 (88.7)	649 (90.2)
	Mix-and-match	42 (9.1)	29 (11.3)	71 (9.8)

The overall crude effectiveness for hospital admission of one dose, two doses and booster vaccination for hospital admission were 24% (95% CI:17–31), 35% (95% CI:29–40) and 27% (95% CI:14–38), respectively. The corresponding value for adjusted effectiveness were 22% (95% CI:14–29), 35% (95% CI:29–41) and 33% (95% CI:16–47), respectively (**Table 3**). The vaccine effectiveness decreases with increasing age. For the 12–44 years age group, the adjusted vaccine effectiveness was 29% (15–42) for one dose, 47% (95% CI:36–57) for two doses, and 44% (95% CI: -5–70) for three doses. However, the corresponding values for age group of 65 years and older were 20% (95% CI: -16–27), 25% (95% CI: 31–56), and 20% (95% CI: 17–46), respectively. The adjusted vaccine effectiveness analysis stratified by specific vaccine brands revealed that for individuals who had received a single dose, the AZD1222 vaccine demonstrated the highest effectiveness, at 37% (95% CI: 22–49). This was followed by the BBIBP-CorV vaccine, which had an effectiveness of 20% (95% CI: 11–28). In addition, the adjusted vaccine effectiveness for homologous primary vaccination was 34% (95% CI: 27–40). Considering the history of infection as equivalent to one dose of the vaccine did not change VE significantly (**Table 3**).

**Table 3:** Crude and adjusted vaccine effectiveness for hospital admission, overall and by age group and by vaccine type.

Variables	Population or exposure or study period	N (19314)	Cases;vacc/Controls; vacc	Crude VE	CI	Adjusted VE*	CI
**All ages**	Partially vaccinated	2443	1587/856	24	17-31	22	14-29
	Fully vaccinated	2797	1718/1079	35	29-40	35	29-41
	Booster vaccinated	720	461/259	27	14-38	33	16-47
Age categories							
**12-44**	Partially vaccinated	554	349/205	31	17-42	29	15-42
	Fully vaccinated	464	273/191	42	29-52	47	36-57
	Booster vaccinated	49	31/18	30	-26- 61	44	-5- 70
**45-64**	Partially vaccinated	1057	723/334	18	5-29	18	5-29
	Fully vaccinated	881	559/322	34	24-44	39	28-48
	Booster vaccinated	195	120/75	40	19-55	46	18-64
**>=65**	Partially vaccinated	831	514/317	22	8-34	20	-16-27
	Fully vaccinated	1452	886/566	25	14-34	25	31-56
	Booster vaccinated	476	310/166	10	-10- 27	20	17-46
All ages							
**One dose**	BBIBP-CorV	1828	1191/637	23	15-31	20	11-28
	AZD1222	415	248/167	39	26-50	37	22-49
	Others	199	147/52	-16	-60-16	-11	-53-20
**Fully vaccinated**	Homologous	2784	1711/1073	35	29-40	34	27-40
	Heterologous	13	7/6	–	–	–	–
**Booster**	Homologous	649	419/230	21	-32-52	-42	-99- 70
	Heterologous	71	42/29	–	–	–	–

In addition, the effectiveness based on the dominant circulating variant is detailed in **Appendix, Table 1**, revealing superior effectiveness during the Delta variant circulation. The efficacy of primary vaccination with AZD1222 outperformed other vaccines during the Delta variant period, with no significant difference observed during the Omicron variant circulation (**Appendix**, **Table 1**).

The crude VE against severe COVID-19 outcomes (death and/or ICU admission) among individuals who received one dose, two doses, and booster shots was 31% (95% CI: 18–43), 31% (95% CI 18–42), and 10% (95% CI -26–35), respectively. The corresponding values for adjusted (for age, sex, province, contact history of a patient with COVID-19, date of first symptoms, length of hospital stays, and time period longer than six months after vaccination) vaccine effectiveness were 33% (95% CI 19- 44), 34% (95% CI 20- 45) and 20% (95% CI -29- 50) (**Table 4**). Considering the first dose, the AZ1222 exhibited comparatively superior protection against severe outcome (VE=47%, 95%CI 18%-66%). Apart from the first dose, subsequent doses of vaccination did not demonstrate significant effectiveness during the circulation of the Omicron variant, as indicated in **Appendix, Table 2**.

**Table 4:** Crude and adjusted vaccine effectiveness for sever COVID, overall and by age group and by vaccine type.

Variables	Population or exposure or study period	N (4483)	Cases; vacc/Controls; vacc	Crude VE	CI	Adjusted VE*	CI
**All ages**	Partially vaccinated	610	383/227	31	18-43	33	19-44
	Fully vaccinated	717	452/265	31	18-42	34	20-45
	Booster vaccinated	174	120/54	10	-26- 35	20	-29-50
Age categories							
**12-44**	Partially vaccinated	107	64/43	39	8- 52	45	16-64
	Fully vaccinated	103	52/51	58	37-72	60	37-75
	Booster vaccinated	13	12/1	–	–	–	–
**45-64**	Partially vaccinated	246	158/88	27	15-53	37	16-53
	Fully vaccinated	198	126/72	38	15- 55	39	16-57
	Booster vaccinated	38	22/16	52	6-75	31	-64-71
**>=65**	Partially vaccinated	257	161/96	20	-8- 40	19	-10-40
	Fully vaccinated	416	274/142	8	-18-28	13	-15-34
	Booster vaccinated	123	86/37	-11	-69- 27	22	-51- 60
All ages							
**One dose**	BBIBP-CorV	466	293/173	31	16-44	32	16-45
	AZD1222	97	56/41	45	16-63	47	18-66
	Others	47	34/13	-7	-99-44	-10	-99-43
**Fully vaccinated**	Homologous	715	451/264	31	18-42	32	17-44
	Heterologous	2	1/1	–	–	–	–
**Booster**	Homologous	155	103/49	39	-77-79	30	-99-78
**-**	Heterologous	22	17/5	–	–	–	–

Sensitivity analyses were conducted in patients who had previous infections with the coronavirus (SARS-CoV-2), considering the history of infection as equivalent to one dose of the vaccine. There were no significant alterations observed in the estimated VEs.

## Discussion

This nationwide test-negative study conducted in Iran is the first of its kind to assess the effectiveness of COVID-19 vaccines. The findings indicate that individuals who received both the primary vaccination and a booster dose experienced a reduction in hospitalizations due to COVID-19, regardless of the vaccine type. Specifically, the vaccination program showed a 35% reduction in hospitalizations during the epidemic with the Delta variant and a 33% reduction during the Omicron variant dominant period. Regardless of the variant circulating at the time, individuals who received full primary vaccination were less likely to experience severe outcomes such as ICU admission and/or death due to COVID-19. The vaccine effectiveness was estimated to be 34% for severe outcomes with full primary vaccination and 20% with the booster dose. No effective protection was observed against severe outcome during circulation of Omicron variant. It is worth noting that the vaccination coverage among the study participants was low, with only 30.8% having received at least one dose of the vaccine.

Compared to a recent national cohort study on ICU admissions for COVID-19, where reported effectiveness was 8%, 20%, and 33% for individuals with the first, second, and booster COVID-19 vaccinations, our study demonstrates greater effectiveness in preventing death during circulation of Delta variant. Nevertheless, the effectiveness of booster doses in our research is observed to be lower than that reported in the mentioned study (19). Our reported effectiveness, however, was lower than the maximum effectiveness observed in Guilan, Iran, against hospitalization. In Guilan, 151 days after receiving the second dose of the BBIBP-CorV vaccine (the main vaccine used in our population), the effectiveness was reported at 85% (95% CI: 77%-91%). Furthermore, for preventing death 91–120 days after the second dose of BBIBP-CorV, the effectiveness was 56% (95% CI: 33%-71%) (7).

The observed differences in reported effectiveness are partly due to the variation in methodology in these three studies. The study by Jamaati, H., et al. (2023) is a classic case-control study on patients admitted to ICUs with the clinical and paraclinical parameters for the diagnosis of COVID-19 (19). They did not clearly define the criteria for the definition of COVID-19. Therefore, the case and control selection in this study is completely different from ours, where the result of the PCR test was the only criteria for classifying the patients as cases or controls. In addition, the final ORs were adjusted for only age, sex, and comorbidities and not for the time since the last vaccination, which is an effective factor influencing the VE.

The study by Heidarzadeh, A., et al. (2023) is a test-negative case-control study with the same diagnosis criteria as we used. However, the control group in ours is different from what they had. They did not use other SARI admitted patients, but those who referred to the public health centers for testing (outpatient setting) (7). Such patients are obviously in better condition with milder signs and symptoms. In addition, the authors did not follow the test-negative case-control design for the calculation of effectiveness against ICU admission and death. To do so, one should restrict the number of participants to those admitted to the ICU and those who died, respectively. Except for such methodological differences in selecting the control group and analysis, both studies followed almost the same strategies for adjustment of confounding variables.

Interestingly, both studies by Heidarzadeh, A. (2023), and ours did not show any effect of vaccination on ICU admission (the results have not been presented). Other Iranian studies did not report or could not report any effect of vaccine on ICU admission (7, 18). However, results from other reports have already shown that, similar to hospital admission and death, the likelihood of ICU admission is effectively reduced after full and booster vaccination with most vaccines (22, 23). The reason for not observing the protective effect of the COVID-19 vaccination on ICU admission is not clear and needs further investigation.

The study by Rosenberg, E., et al. (2022) assessed the effectiveness of the three COVID-19 vaccines authorized in the US against infection and hospitalization in New York. Vaccine effectiveness declined over time as the Delta variant became predominant, dropping from over 90% to around 70%. However, effectiveness against hospitalization remained high, with modest declines only seen in older adults vaccinated with Pfizer or Moderna. The Ad26.COV2.S (Johnson & Johnson) vaccine had lower overall effectiveness compared to the mRNA vaccines (12). Lopez Bernal, J,. et al. (2021) founded that vaccine effectiveness against the Delta variant (30.7%) was lower compared to the Alpha variant (48.7%), especially after a single vaccine dose. However, with two doses, BNT162b2 vaccine’s effectiveness was 93.7% against Alpha and 88.0% against Delta, while ChAdOx1 nCoV-19 vaccine’s was 74.5% against Alpha and 67.0% against Delta (13).

Andrews, N,. et al. (2022) assessed vaccine effectiveness against the Omicron and Delta variants in England. Vaccine effectiveness was higher against the Delta variant compared to Omicron at all-time points. After two doses of the ChAdOx1 nCoV-19 vaccine, no effect was seen against Omicron from 20 weeks onward, while two doses of BNT162b2 had 65.5% effectiveness at 2–4 weeks, dropping to 8.8% at 25+ weeks. A BNT162b2 or mRNA-1273 booster after the primary series increased protection, but this also waned over time, reaching 45.7% and 60.9% respectively at 10+ weeks (14).

The lower vaccine effectiveness observed in our study compared to other reports from elsewhere can be attributed to several factors. Firstly, the predominant use of inactivated vaccines in Iran, which generally have lower effectiveness compared to mRNA vaccines. Additionally, the delayed start of the COVID-19 vaccination program in Iran, beginning 11 months after the first case, and the slow rate of vaccination contributed to lower vaccine effectiveness because of depletion of susceptible. Furthermore, in contrast to previous studies in Iran, our research involved a larger sample size during the high prevalence of the Omicron variant, recognized for its heightened resistance to current vaccines. In fact, our results showed no protective effect of vaccination against severe outcome when the dominant variant of circulation was Omicron. The prolonged recruitment period for cases and controls coincided with the easing of restrictive measures designed to curb COVID-19 transmission, such as school closures, mask mandates, and various public health interventions. These collective factors contributed to a reduction in the estimated vaccine effectiveness in our study.

Our study is the first nationwide study on the effectiveness of COVID-19 vaccines in Iran. In fact, the selection of cases and controls was based on the international guidelines (24). However, we did not recruit cases and controls in each hospital by the time they were admitted. Instead, we used data registration. Therefore, the misclassification of cases and controls because of the false negative result of the RT-PCR test is likely. In fact, the rate of false negative tests ranges from 1% to 30% (25, 26) and such results might be due to suboptimal specimen collection, testing too early in the disease process, inappropriate specimen type and variability in viral shedding (27). It is difficult to estimate the exact direction of the effect of such misclassification, but it is most likely to be non-dependent and non-differential and therefore underestimate the overall effect of vaccination. Another misclassification that might contribute to errors in our estimates of vaccine effectiveness is due to the precision of the vaccination record. Sensitivity and specificity of such data might be different by province, and unfortunately, we have no such report in order to correct our estimate for the misclassification of vaccination status. However, even a very high sensitivity (96%) and specificity (90%) of the vaccination record causes a 4%-6% decrease in estimated vaccine effectiveness. The combination of such misclassifications in our exposure plus the outcome may even affect our estimated effectiveness further. In addition, in our case-control study, the number of cases is twice that of controls. The best scenario for a case-control study is having an equal number of cases and controls. However, in epidemic situations, it sometimes happens that there are not sufficient controls, which might decrease the power of the study.

## Conclusion

In conclusion, despite the lower effectiveness of inactivated vaccines (at least for hospital admission), our study showed that full vaccination provided relative advantages in preventing hospital admissions and deaths due to COVID-19 in Iran. This effect was more pronounced during the circulation of the Delta variant and decreased significantly during the dominance of the Omicron variant. The substantial disparities between the estimated effectiveness within our real-world data and the findings reported in randomized controlled trials underscore the importance of ongoing assessments of vaccine performance in populations that frequently encounter new variants, evolving public policies, and shifts in individual behaviors.

## Data Availability

The original contributions presented in the study are included in the article/**Supplementary Material**. Further inquiries can be directed to the corresponding author.

## References

[1] Kim JH Marks F Clemens JD. Looking beyond COVID-19 vaccine phase 3 trials. Nat Med. (2021) 27:205–11. doi: 10.1038/s41591-021-01230-y 33469205

[2] Ripabelli G Sammarco ML Rezza G D’Amico A De Dona R Iafigliola M. A SARS-CoV-2 outbreak among nursing home residents vaccinated with a booster dose of mRNA COVID-19 vaccine. J Community Health. (2022) 47:598–603. doi: 10.1007/s10900-022-01082-8 35334031 PMC8949830

[3] Haas EJ Angulo FJ McLaughlin JM Anis E Singer SR Khan F. Impact and effectiveness of mRNA BNT162b2 vaccine against SARS-CoV-2 infections and COVID-19 cases, hospitalisations, and deaths following a nationwide vaccination campaign in Israel: an observational study using national surveillance data. Lancet. (2021) 397:1819–29. doi: 10.1016/S0140-6736(21)00947-8 PMC8099315 33964222

[4] Polack FP Thomas SJ Kitchin N Absalon J Gurtman A Lockhart S. Safety and efficacy of the BNT162b2 mRNA Covid-19 vaccine. New Engl J Med. (2020) 383:2603–15. doi: 10.1056/NEJMoa2034577 PMC7745181 33301246

[5] Baden LR El Sahly HM Essink B Kotloff K Frey S Novak R. Efficacy and safety of the mRNA-1273 SARS-CoV-2 vaccine. New Engl J Med. (2021) 384:403–16. doi: 10.1056/NEJMoa2035389 PMC7787219 33378609

[6] Palacios R Batista AP Albuquerque CSN Patiño EG Santos JdP Tilli Reis Pessoa Conde M. Efficacy and safety of a COVID-19 inactivated vaccine in healthcare professionals in Brazil: the PROFISCOV study. (2021).

[7] Heidarzadeh A Moridani MA Khoshmanesh S Kazemi S Hajiaghabozorgi M Karami M. Effectiveness of COVID-19 vaccines on hospitalization and death in Guilan, Iran: a test-negative case-control study. Int J Infect Dis. (2023) 128:212–22. doi: 10.1016/j.ijid.2022.12.024 PMC9788848 36572376

[8] Andrews N Tessier E Stowe J Gower C Kirsebom F Simmons R. Vaccine effectiveness and duration of protection of Comirnaty, Vaxzevria and Spikevax against mild and severe COVID-19 in the UK. medrxiv. (2021). doi: 10.1101/2021.09.15.21263583

[9] Tenforde MW Self WH Naioti EA Ginde AA Douin DJ Olson SM. Sustained effectiveness of Pfizer-BioNTech and Moderna vaccines against COVID-19 associated hospitalizations among adults—United States, March–July 2021. Morbidity Mortality Weekly Rep. (2021) 70:1156. doi: 10.15585/mmwr.mm7034e2 PMC8389395 34437524

[10] Chemaitelly H Tang P Hasan MR AlMukdad S Yassine HM Benslimane FM. Waning of BNT162b2 vaccine protection against SARS-CoV-2 infection in Qatar. New Engl J Med. (2021) 385:e83. doi: 10.1056/NEJMoa2114114 34614327 PMC8522799

[11] Cevik M Grubaugh ND Iwasaki A Openshaw P. COVID-19 vaccines: Keeping pace with SARS-CoV-2 variants. Cell. (2021) 184:5077–81. doi: 10.1016/j.cell.2021.09.010 PMC8445744 34534444

[12] Rosenberg ES Dorabawila V Easton D Bauer UE Kumar J Hoen R. Covid-19 vaccine effectiveness in New York state. New Engl J Med. (2022) 386:116–27. doi: 10.1056/NEJMoa2116063 PMC8693697 34942067

[13] Lopez Bernal J Andrews N Gower C Gallagher E Simmons R Thelwall S. Effectiveness of Covid-19 vaccines against the B. 1.617. 2 (Delta) variant. New Engl J Med. (2021) 385:585–94. doi: 10.1056/NEJMoa2108891 PMC8314739 34289274

[14] Andrews N Stowe J Kirsebom F Toffa S Rickeard T Gallagher E. Covid-19 vaccine effectiveness against the Omicron (B. 1.1. 529) variant. New Engl J Med. (2022) 386:1532–46. doi: 10.1056/NEJMoa2119451 PMC8908811 35249272

[15] Pilishvili T Gierke R Fleming-Dutra KE Farrar JL Mohr NM Talan DA. Effectiveness of mRNA Covid-19 vaccine among US health care personnel. New Engl J Med. (2021) 385:e90. doi: 10.1056/NEJMoa2106599 34551224 PMC8482809

[16] He X Su J Ma Yn Zhang W Tang S. A comprehensive analysis of the efficacy and effectiveness of COVID-19 vaccines. Front Immunol. (2022) 13:945930. doi: 10.3389/fimmu.2022.945930 36090988 PMC9459021

[17] What share of the population has received at least one dose, and completed the initial vaccination protocol?(2023). Available online at: https://ourworldindata.org/covid-vaccinations.

[18] Mirahmadizadeh A Heiran A Bagheri Lankarani K Serati M Habibi M Eilami O. editors. "Effectiveness of coronavirus disease 2019 vaccines in preventing infection, hospital admission, and death: a historical cohort study using Iranian registration data during vaccination program". In: Open Forum Infectious Diseases. Oxford University Press US (2022). doi: 10.1093/ofid/ofac177 PMC9126490 35615300

[19] Jamaati H Karimi S Ghorbani F Panahi Y Hosseini‐Baharanchi FS Hajimoradi M. Effectiveness of different vaccine platforms in reducing mortality and length of ICU stay in severe and critical cases of COVID-19 in the Omicron variant era: A national cohort study in Iran. J Med Virol. (2023) 95:e28607. doi: 10.1002/jmv.28607 36815507

[20] Organization WH. Estimating COVID-19 vaccine effectiveness against severe acute respiratory infections (SARI) hospitalisations associated with laboratory-confirmed SARS-CoV-2: an evaluation using the test-negative design: guidance document. Regional Office for Europe: World Health Organization (2021).

[21] WHO. WHO surveillance case definitions for ILI and SARI(2018). Available online at: http://www.who.int/influenza/surveillance_monitoring/ili_sari_surveillance_case_definition/en.

[22] Jara A Undurraga EA Zubizarreta JR González C Pizarro A Acevedo J. Effectiveness of homologous and heterologous booster doses for an inactivated SARS-CoV-2 vaccine: a large-scale prospective cohort study. Lancet Global Health. (2022) 10:e798–806. doi: 10.1016/S2214-109X(22)00112-7 PMC9034854 35472300

[23] Fu Y Zhao J Wei X Han P Yang L Ren T. Effectiveness and cost-effectiveness of inactivated vaccine to address COVID-19 pandemic in China: evidence from randomized control trials and real-world studies. Front Public Health. (2022) 10:917732. doi: 10.3389/fpubh.2022.917732 35928479 PMC9343737

[24] ECfDPa. C. Core protocol for ECDC VEBIS studies of COVID-19 vaccine effectiveness against hospitalisation with Severe Acute Respiratory Infection laboratory-confirmed with SARS-CoV-2 or seasonal influenza. In: ECDC editor (2023).

[25] Arevalo-Rodriguez I Buitrago-Garcia D Simancas-Racines D Zambrano-Achig P Del Campo R Ciapponi A. False-negative results of initial RT-PCR assays for COVID-19: A systematic review. PloS One. (2020) 15:e0242958. doi: 10.1371/journal.pone.0242958 33301459 PMC7728293

[26] Long DR Gombar S Hogan CA Greninger AL O'Reilly-Shah V Bryson-Cahn C. Occurrence and timing of subsequent severe acute respiratory syndrome coronavirus 2 reverse-transcription polymerase chain reaction positivity among initially negative patients. Clin Infect Dis. (2021) 72:323–6. doi: 10.1093/cid/ciaa722 PMC7314163 33501950

[27] Kanji JN Diggle M Bulman DE Hume S Taylor S Kelln R. Retrospective testing of respiratory specimens for COVID-19 to assess for earlier SARS-CoV-2 infections in Alberta, Canada. J Assoc Med Microbiol Infect Dis Can. (2021) 6:10–5. doi: 10.3138/jammi-2020-0035 PMC9612433 36340216

