# Supplementary material for: Evaluation of the COVID-19 vaccine effectiveness on the outcomes of COVID 19 disease in Iran: a test-negative case-control study
Source: Front Immunol. 2024 Aug 20;15:1420651. doi: 10.3389/fimmu.2024.1420651 (PMC11372784; doi:10.3389/fimmu.2024.1420651)
Supplement: Supplementary file 1 [file DataSheet1.pdf]

Appendix 1, Table 1. *Crude and Adjusted Vaccine Effectiveness for hospital admission against COVID-19, overall and by age group and by vaccine type, and peak type*

		Delta (from 2021/30/4 to 2021/31/12)						Omicron (from 2022/1/1 to 2022/20/6)					
**Variables**	**Population or exposure or study period**	**N (4,261)**	**Cases;vacc / Controls; vacc**	**Crude VE**	**CI**	**Adjusted VE***	**CI**	**N (1698)**	**Cases;vacc / Controls; vacc**	**Crude VE**	**CI**	**Adjusted VE***	**CI**
**All ages**	Partially vaccinated	2,299	1506/793	23	15-30	20	12-28	144	81/63	31	1-53	39	10-58
	Fully vaccinated	1,938	1175/763	38	31-46	33	26-40	585	542/316	9	-14 -27	25	3-42
	Booster vaccinated	24	11/13	66	33-85	52	-13- 80	696	450/246	2	-23- 23	30	3- 49
**Age categories**													
**12-44**	Partially vaccinated	507	320/187	31	16-43	28	13- 41	47	29/18	19	-57 -51	23	-56- 62
	Fully vaccinated	199	104/95	56	41-67	59	44-70	265	169/96	12	-31- 41	30	-11- 56
	Booster vaccinated	1	1/0	-	-	-	-	48	169/18	16	-62- 57	32	-42- 68
**45-64**	Partially vaccinated	1022	708/314	16	3-28	15	0-27	35	15/20	55	4-88	63	20- 83
	Fully vaccinated	619	395/224	35	22-45	36	23-47	262	164/98	-2	-52- 32	23	-22- 52
	Booster vaccinated	8	2/6	88	39-98	88	37-98	287	118/69	-4	-60- 32	27	-3- 59
**>=65**	Partially vaccinated	770	478/292	22	7-44	19	3-32	62	37/25	25	-34- 59	30	-29- 62
	Fully vaccinated	1120	676/444	27	16-37	21	8-37	331	209/122	14	-24- 40	34	-5- 59
	Booster vaccinated	15	8/7	45	-52- 81	3	-99- 68	461	302/159	4	-35- 32	35	-12- 62
**All ages**													
**One dose**	BBIBP-CorV	1722	1133/589	22	13-30	18	8-27	106	58/48	36	2-58	54	25-72
	AZD1222	390	232/158	41	27-52	38	23-50	25	16/9	5	-99-59	55	-22- 83
	Others	187	141/46	-25	-74-11	-19	-67-16	12	6/6	47	-68- 93	63	-26- 90
**Fully vaccinated**	BBIBP-CorV	1589	974/615	36	29-42	32	24-40	621	392/229	9	-16-28	23	-2-42

	AZD1222	160	87/73	52	34-65	46	24-62	153	98/55	5	-38-35	23	-24-52
	Others	189	114/75	38	17-56	39	17-55	85	53/32	12	-42-45	37	-8-63
**Booster**	Homologue	18	10/8	-	-	-	-	631	409/222	-7	-29- 2	8	-16- 28
	Heterologous	6	1/5	-	-	-	-	65	41/24	1	-66-41	10	-55- 48

***Adjusted with age, sex, province, contact history of patient with COVID-19, days between onset of symptom and swabbing, time from vaccination to admission (>6 months)**

Appendix 1, Table 2. *Crude and Adjusted Vaccine Effectiveness for sever COVID-19, overall and by age group and by vaccine type, and peak typ*

		Delta (from 2021/30/4 to 2021/31/12)						Omicron (from 2022/1/1 to 2022/20/6)					
**Variables**	**Population or exposure or study period**	**N (3971)**	**Cases;vacc / Controls; vacc**	**Crude VE**	**CI**	**Adjusted VE***	**CI**	**N (512)**	**Cases;vacc / Controls; vacc**	**Crude VE**	**CI**	**Adjusted VE***	**CI**
**All ages**	Partially vaccinated	573	365/208	29	15-42	31	16-43	37	18/19	52	-3-78	69	10-90
	Fully vaccinated	526	331/195	32	17-44	32	16-55	191	121/70	12	-44-46	27	-46-63
	Booster vaccinated	5	2/3	73	-62- 96	74	-78- 96	169	118/51	-18	-97-29	-1	-99-56
**Age categories**													
**12-44**	Partially vaccinated	103	62/41	38	6-59	45	14- 64	4	2/2	-	-	-	-
	Fully vaccinated	55	22/33	73	53-85	79	60-89	48	30/18	17	-99-70	9	-99-79
	Booster vaccinated	0	0	-	-	-	-	13	12/1	-	-	-	-
**45-64**	Partially vaccinated	234	155/79	31	7- 49	30	3- 49	12	3/9	-	-	-	-
	Fully vaccinated	145	96/49	31	0-53	31	-2- 54	53	30/23	41	-49-77	54	-59-87
	Booster vaccinated	1	0/1	-	-	-	-	37	22/15	34	-80-76	59	-99-92
**>=65**	Partially vaccinated	236	148/88	21	-8- 42	19	-11- 41	21	13/8	-	-	-	-
	Fully vaccinated	326	213/113	11	-18- 33	7	-25- 31	90	61/29	-16	-99-63	50	-95-87
	Booster vaccinated	4	2/2	53	-99- 94	51	-99- 3	119	84/35	-33	-99-32	32	-99-85
**All ages**													
**One dose**	BBIBP-CorV	440	281/159	29	12-42	30	12-43	26	12/14	-	-	-	-
	AZD1222	92	52/40	48	20-66	49	21-68	5	4/1	-	-	-	-
	Others	41	32/9	-44	-99-32	-44	-99-32	6	2/4	-	-	-	-

**Fully vaccinated**	BBIBP-CorV	429	280/149	24	6-39	26	6-42	128	80/48	3	-57-41	0	-94-48
	AZD1222	42	24/18	46	0-71	45	-8-72	47	34/13	-56	--99-25	-86	-99-37
	Others	55	27/28	61	34-78	62	34-78	16	7/9	-		-	-
**Booster**	Homologue	4	2/2	-	-	-	-	148	101/47	-27	-92-16	-42	-99-19
	Heterologous	1	0/1	-	-	-	-	21	17/4	-	-	-	-

***Adjusted with age, sex, province, contact history of patient with COVID-19, days between onset of symptom and swabbing, time from vaccination to admission (>6 months)**
